# The Adsorption Efficiency of Regenerable Chitosan-TiO_2_ Composite Films in Removing 2,4-Dinitrophenol from Water

**DOI:** 10.3390/ijms24108552

**Published:** 2023-05-10

**Authors:** Jennifer Gubitosa, Vito Rizzi, Paola Fini, Sergio Nuzzo, Pinalysa Cosma

**Affiliations:** 1 Department of Chemistry, University of Bari “Aldo Moro”, Via Orabona, 4-70126 Bari, Italy; jennifer.gubitosa@uniba.it; 2 National Research Council, Institute for Chemical and Physical Processes, CNR-IPCF, Via Orabona, 4-70126 Bari, Italy; p.fini@ba.ipcf.cnr.it (P.F.); sergio.nuzzo@ba.ipcf.cnr.it (S.N.)

**Keywords:** chitosan film, TiO_2_, 2,4-dinitrophenol, photodegradation, adsorption, water pollution, advanced oxidation processes

## Abstract

In this work, the great performance of chitosan-based films blended with TiO_2_ (CH/TiO_2_) is presented to adsorb the hazardous pollutant 2,4-dinitrophenol (DNP) from water. The DNP was successfully removed, with a high adsorption %: CH/TiO_2_ exhibited a maximum adsorption capacity of 900 mg/g. For pursuing the proposed aim, UV–Vis spectroscopy was considered a powerful tool for monitoring the presence of DNP in purposely contaminated water. Swelling measurements were employed to infer more information about the interactions between chitosan and DNP, demonstrating the presence of electrostatic forces, deeply investigated by performing adsorption measurements by changing DNP solutions’ ionic strength and pH values. The thermodynamics, adsorption isotherms, and kinetics were also studied, suggesting the DNP adsorption’s heterogeneous character onto chitosan films. The applicability of pseudo-first- and pseudo-second-order kinetic equations confirmed the finding, further detailed by the Weber–Morris model. Finally, the adsorbent regeneration was exploited, and the possibility of inducing DNP desorption was investigated. For this purpose, suitable experiments were conducted using a saline solution that induced the DNP release, favoring the adsorbent reuse. In particular, 10 adsorption/desorption cycles were performed, evidencing the great ability of this material that does not lose its efficiency. As an alternative approach, the pollutant photodegradation by using Advanced Oxidation Processes, allowed by the presence of TiO_2_, was preliminary investigated, opening a novel horizon in the use of chitosan-based materials for environmental applications.

## 1. Introduction

Water pollution is one of the major global concerns for our society because it can induce risks both for humans and aquatic life [1,2]. Different pollutants are poured daily into the water: heavy metals, aromatic or aliphatic compounds, hydrocarbons, amines, textile dyes, and emerging pollutants are only some examples. A few of these chemicals are endocrine disruptors, carcinogenic, mutagenic, etc. [3]. In many cases, these hazardous materials, even at very low concentrations, could hardly impact human health, highlighting the importance of their removal from water [3,4,5,6,7]. Among pollutants, aromatic compounds such as phenols, mainly arising from industrial wastewater, should have attention paid to them due to their deleterious effects [8,9,10,11,12]. These pollutants are very toxic and harmful to the environment, and, unfortunately, a huge amount of these substances, or their derivatives, are retrieved in water, being usually used to synthesize insecticides, dyes, polymers, rubber chemicals, pigments, drugs, and wood preservatives [8].

2,4-dinitrophenol (DNP) is one of the main persistent organic phenols detected in industrial effluent streams, and it represents a danger, even in low amounts. Indeed, DNP has low biodegradability, high solubility, and stability [8]. DNP could favor endocrine and reproductive system problems, eye and skin irritation, headache, hepatic lesions, irregular heartbeat, central nervous system damage, eczema, nausea, vertigo, inhibition of cell growth, cardiovascular diseases, and carcinogenesis [9,10]. Not surprisingly, the United States Environmental Protection Agency reports DNP as a priority pollutant, advising its amount in natural water lower than 10 ng/L [10]. Therefore, finding an effective method for DNP removal from the environment, and reducing its amount, at least to the extent permitted by law, should be considered a main concern. However, the challenge for researchers in this field is finding simple removal processes that are effective and inexpensive. For example, chemical oxidation, microbial degradation, photocatalytic degradation, electrochemical degradation, solvent-based extraction, and membrane separation are reported as the main strategies for DNP removal from water [11,12]. Nevertheless, these practices could exhibit negative aspects, such as large production of wastes, high use of electric current and chemicals, low efficiencies in removing the pollutant, regeneration of toxic by-products, and high costs. On the other hand, adsorption techniques have received considerable attention for wastewater remediation due to the usually associated low cost, simplicity of design, strong regeneration performance, and ease of operation, conferring a definite possibility to be applied at the industrial level [8,13,14,15,16,17,18,19,20,21]. Currently, synthetic and natural adsorbents are listed in the literature for DNP removal from water (Table 1). For example, Krishnan et al. [14] used active carbon exhibiting a q_max_ of 277.78 mg/g, and the favorable pH range for the adsorption process was found to be 2.0–5.0. Bettini et al. [15] reported a 58.66% removal efficiency at pH 6.4 when using SiO_2_-based nanocomposite. Walia et al. [16] studied an amine-functionalized metal–organic framework showing the 99% removal of 2,4-DNP in 50 min at pH 4. In this case, the adsorbent’s recyclability was assessed using an organic solvent, such as ethanol. The removal efficiencies were found to be 97%, 89%, 79%, 65%, and 52% after five cycles, respectively. So, the performance of the material was reduced after several uses. Ultrasound-assisted magnetic adsorption graphene oxide-Fe_3_O_4_-based system was proposed by Azari et al. [17]. The material showed a relatively high adsorption capacity q_max_ towards DNP, reporting a value of 425.58 mg/g. A polymer obtained by loading ionic liquids on silica was prepared by Cheng et al. [8] with a maximum adsorption capacity of 114.7 mg/g. Ismail et al. [18] used sol–gel Titania-silica-mixed imidazolium-based ionic liquid having a very low maximum adsorption capacity of 7.78 mg/g. Magdy et al. [19] studied char ash, retrieving a maximum adsorption capacity of 7.55 mg/g. Thang et al. [20] proposed the use of chicken manure biochar. The adsorption capacity, in this case, was 148.1 mg/g, and the adsorbent showed high reuse ability five times, but the % of removal collapsed after prolonged use. Moreover, C_2_H_5_OH was proposed for the adsorbent’s regeneration, working far from eco-friendly approaches.

As a result, although different adsorbents were proposed to remove DNP, the retrieved adsorption capacities were relatively low. Moreover, the costs associated with the adsorbent were not low in many cases. Additionally, the possibility of reusing the material without affecting the removal performance, according to sustainability and Green Chemistry principles, has not yet been fully explored [21]. Hence, the key to solving this problem is to develop long-lasting recyclable and low-cost adsorbents with high adsorption capacities that can quickly depurate water from DNP. This paper would thus face this problem by proposing free-standing chitosan hybrid films not yet investigated in the past literature for DNP removal, which is able to remove it from water in a few minutes with a very high adsorption capacity (q_max_: 900 mg/g, see also Table 1), offering at the same time the possibility to regenerate the adsorbent. Indeed, DNP desorption using a diluted NaCl solution allowed the pollutant and adsorbent recovery at least 10 times. Interestingly, although 10 cycles of adsorption and desorption were accomplished, the chitosan film performance remained the same, supporting the long-time usage of the adsorbent, thus suggesting the potential scale-up from laboratory to industrial scale.

An alternative approach for reusing chitosan films was preliminarily investigated during this work to show the additional value of the proposed adsorbent. Specifically, DNP solid-state photodegradation through Advanced Oxidation Processes (AOPs), enabled by the embedded commercial photocatalyst TiO_2_, was studied. The aim was to show an additional way to favor adsorbent recycling, preventing pollutant recovery. Some preliminary experiments were thus attempted to point out the feasibility of this approach being worth investigating in the future for elucidating the mechanisms and kinetics of the involved reactions. However, hybrid materials based on different photocatalysts were largely employed in the past literature for polyphenol removal [22,23].

Interestingly, using TiO_2_ offers several advantages: it shows low toxicity, chemical stability, availability, convenient physical and optical properties, and it is considered low cost. As reported by Sescu et al. [24], TiO_2_-based heterogeneous photocatalysis is described as an efficient technology for the removal of different organic pollutants from water [24]. Specifically, TiO_2_ shows benefits in the photodegradation of pollutants, producing highly reactive radical species and destroying without selectivity the contaminants. If focusing on DNP removal and photodegradation, some examples from the recent literature are worth mentioning and are reported in Table 2 [25,26,27,28,29,30,31].

The number of works devoted to photocatalysis and also based on the use of hybrid materials is large. The list of examples reported in Table 2 could be enlarged, suggesting that the results obtained during this work could offer a valid alternative strategy and a starting point for recycling the adsorbent. Moreover, using a hybrid composite based on chitosan, a low-cost natural biopolymer derived from seafood wastes, would respect the Green Chemistry and Circular Economy principles [32,33,34,35,36,37,38]. Another advantage of this work could be that the TiO_2_ was physically blocked inside the film, avoiding its release/recovery in/from water. Indeed, the dispersion in water of TiO_2_ could negatively affect the ecosystem [39]. Furthermore, chitosan films were largely used for water remediation, being able to remove different classes of compounds, as discussed in the literature [32,33,34,35,36,37,38]. Accordingly, our previous papers [22,23,24,25] also show that the proposed adsorbent removes different families of pollutants in mixtures. Hence, this work would enlarge the chitosan films’ applicability, still nowadays an object of study, by considering the actual literature, opening novel horizons into the environmental field.

## 2. Results and Discussion

### 2.1. An Overview

**Figure 1:**
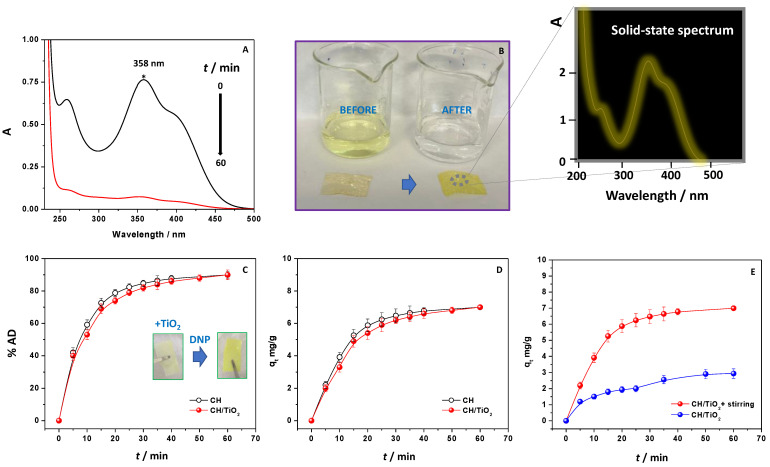
UV–Vis spectra of a DNP solution registered before and after the contact with CH film (for 60 min) (**A**); camera pictures of water polluted with DNP before and after its removal, with corresponding CH film, before and after DNP adsorption (**B**); % of DNP adsorption (**C**) and the corresponding q_t_ values (**D**) calculated for CH and CH/TiO_2_; q_t_ values of CH/TiO_2_ referred to experiments of DNP removal performed in the presence and absence of stirring (**E**). The zoom of (**B**) is related to the solid-state UV–Vis absorption spectrum of CH+DNP. The inset of (**C**) reports the camera picture of CH/TiO_2_ before and after DNP removal.

For monitoring DNP removal from water, UV–Vis absorption spectroscopy was used. Specifically, the broad absorption band centered at λ 358 nm referred to DNP water solution was considered diagnostic for this purpose (Figure 1A) [40]. Indeed, DNP has a typical absorption signal in the UV–Vis range that confers a characteristic bright yellow color to contaminated water, as illustrated in Figure 1B. In the used solution condition pH, DNP was mainly present in phenolate form, so this band is attributable to an n-π* electronic transition involving the lone pair electrons of oxygen and nitrogen atoms in the chemical structure [41,42]. Since chitosan was used in a blend with TiO_2_, initially, adsorption experiments were performed using CH films without TiO_2_ to demonstrate that the latter had only a photocatalytic activity, not perturbing the adsorption efficiency. In detail, a CH film of 1.0 × 2.0 cm was immersed in contaminated water (DNP amount = 18.50 mg/L, pH 4.5) under constant stirring at r.t. The absorption intensity of DNP was reduced to almost zero in 60 min (Figure 1A), indicating that, through adsorption, CH can remove ≈90% of DNP.

The great efficiency of the process can be clearly appreciated if looking at the water solution that, after contact with the CH film, changed its color from yellow to transparent (Figure 1B), and at the same time, the CH film removed from DNP aqueous solution acquired a brilliant yellow color. The UV–Vis spectroscopic analysis on the solid-state film (zoom in Figure 1B) confirmed the presence of DNP inside the film by showing the typical DNP spectroscopic features.

Then, the nanocomposite CH/TiO_2_ was investigated. Specifically, for comparison, an aliquot of the solution containing DNP in contact with the adsorbents (CH and CH/TiO_2_, 1.0 × 2.0 cm) was collected every 5 min and subjected to UV–Vis analysis. The % of adsorption was thus calculated at each contact time (through Equation (1)) and reported in Figure 1C. The results were the same for both adsorbents and leveled off at 90% within 60 min. The adsorption capacities q_t_ were subsequently calculated using Equation (2) and reported in Figure 1D. The findings demonstrated that the presence of TiO_2_ did not alter the observed efficiency of CH film, which remained still high. Accordingly, CH/TiO_2_ changed its color from white (due to the presence of TiO_2_) to yellow, as previously observed for the bare CH film. Moreover, in this case, the spectroscopic analysis on the solid-state film was performed to point out (i) the absence of changes in the titania absorption spectrum and (ii) the presence of DNP. Figure S1 shows the obtained results. Measurements were performed by using, in this case, the reflectance spectroscopy. According to the literature [43], the results show that CH/TiO_2_ composite exhibited a strong reflectance as observed for TiO_2_ at the wavelength region of 350–400 nm. So, it showed that the band gap energy of the TiO_2_, physically embedded inside CH, was the same being not affected by the chitosan network. Once again, after DNP adsorption, its contribution and presence were confirmed by observing the signals at around 230 nm (see the dotted circle in the Figure S1) and in the wavelength region between 300 and 500 nm (see also the absorption spectrum reported in Figure 1 to understand the finding better).

After these assessments, experiments were performed to infer the CH/TiO_2_ maximum adsorption (q_max_) capacity towards DNP. Specifically, the pollutant removal was monitored until the adsorbent saturation, retrieving a very high q_max_ value of 900 mg/g. Starting from this background, CH/TiO_2_ was subjected to further investigation to point out the main feature of DNP adsorption, the possibility of recycling both chitosan and DNP, and, finally, to preliminary show the solid-state degradation of the adsorbed pollutant to propose an alternative way to reuse the adsorbent.

### 2.2. Effect of Stirring on the Adsorption Process

Solution stirring is an important parameter in adsorption phenomena because it affects the solute/adsorbate distribution in the solution bulk and the formation of the external boundary film [43]. In order to analyze the stirring effect in adsorption behavior, a 1.0 × 2.0 cm CH/TiO_2_ was thus placed in contaminated water (DNP amount = 18.50 mg/L, pH 4.5), at r.t, and monitored for 60 min both in the absence and presence of constant speed stirring. The adsorption capacity values plotted as a function of time are reported in Figure 1E.

It is evident that, in the absence of stirring, the q_t_ values were much lower. Overall, it is well known that the solid-liquid adsorption process depends on time and includes at least three main steps: external mass transfer (transport of the adsorbate from the bulk solution to the liquid film around the adsorbent, named film diffusion), internal diffusion of solute from the adsorbent film surface into the pores (intraparticle diffusion), and surface diffusion and adsorption (physical or chemical) on active sites. Regarding intraparticle diffusion, the process can depend on diffusion in pore volume, surface diffusion, or both mechanisms [44,45,46,47]. In this context, there are many theoretical models to characterize and describe the adsorption mechanism and to find the rate-determining step, from the standard Homogeneous Surface Diffusion Model (HSDM) to the much more complete Pore volume and Surface Diffusion Model (PVSDM) [44,45,47,48]. In general, it is considered that the third step is a rapid and equilibrium step in comparison to the first two ones, likely due to instantaneous equilibrium establishment between adsorbate in solution and adsorbate adsorbed on the adsorbent surface. Thus, the adsorption process could be mainly controlled by the other two types of mass transfer resistance, external and internal diffusion [44,45,47,48].

The results in Figure 1E suggest that the solution agitation incremented the turbulence and dissipation of energy in the mixing area, resulting in reduced boundary layer thickness and facilitating the external mass transfer. In particular, when referring to experimental data reported in Figure 1E, a rough estimation of the mass transfer coefficient (k_emt_) was evaluated by applying the equation proposed by Furusawa and Smith [49]:

$${\left[\frac{d}{dt}\left(\frac{C_{t}}{C_{0}}\right)\right]}_{t=0}=-\frac{mSk_{emt}}{V}$$

where m = chitosan film weight in g (0.033 g), S = external surface area per mass of adsorbent in cm^2^/g (60.61 cm^2^/g), and V = volume of solution examined in L (0.015 L) [45,50,51]. The right term of the equation, instead, represented the slope of the concentration decay plot at t = 0 and was estimated by using the first two data points of the curve at t = 0 and t = 5 min (data not shown). The calculated values obtained were 0.067 cm/s for the sample without stirring and 0.217 cm/s for the sample under stirring. As expected, since the mass transfer influenced DNP adsorption onto chitosan films, the agitation determined the increase in the external mass transfer coefficient, suggesting that an incremented system mobility due to the solution stirring facilitates the DNP transfer across the external layer [48,52]. According to the theory that with increasing agitation, the fluid velocity enhances and the boundary layer diminishes, the trend shown by obtained k_emt_ was in accordance with the previous literature related to adsorption on chitosan films [48,53].

Thus, during this work, all the experiments were performed under constant stirring to ensure an enhanced DNP removal.

### 2.3. Effect of DNP Concentration and CH/TiO_2_ Amount on the Adsorption Process

**Figure 2:**
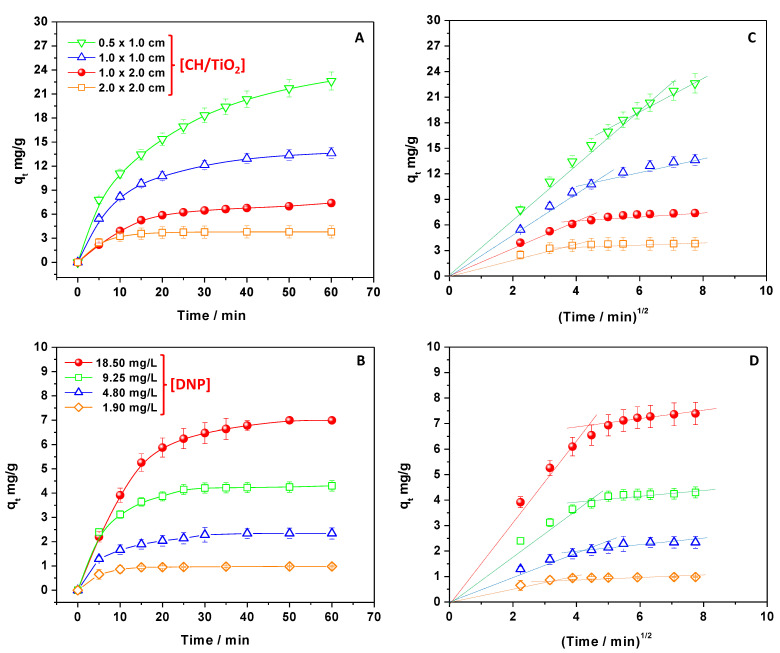
q_t_ values of CH/TiO_2_ calculated by changing the amount of the adsorbent (**A**) and adsorbate (**B**); the Weber–Morris plot applied to data reported in panels A and B (**C**,**D**).

CH/TiO_2_ having different sizes and weights were employed to reveal the adsorbent amount’s role. Under these work conditions, the DNP concentration was fixed at 18.50 mg/L, pH 4.5, and r.t. (Figure 2A). The adsorption capacities were thus calculated to compare the obtained results. It was observed that the q_t_ values decreased at the increase in the adsorbent amount. The finding can be interpreted by looking at the mathematical expression of Equation (2), where the adsorbent’s mass is at the fraction’s denominator. However, when the adsorbent was in excess, the adsorption sites cannot be saturated, thus reducing the whole q_t_ values. In any case, the removal of DNP was almost complete [7,35,54].

Not surprisingly, the plateau region beginning, where the system reached the relative maximum adsorption capacity, occurred in a shorter time when CH/TiO_2_ films of higher dimensions were present [7,35,54]. Indeed, when passing from 2.0 × 2.0 to 0.5 × 1.0 cm film size, the time necessary to join the plateau region changed from 10 to about 60 min. The finding suggested that DNP removal was favored by the adsorbent surface availability, evidencing the role of free active sites during the adsorption [11]. Indeed, at the beginning of the process, the removal of DNP was very fast. Conversely, with the passing of the contact time, the growth of q_t_ values was slower by denoting the reduced number of available sites and the presence of repulsive forces between DNP molecules free in solution and those adsorbed [20]. Therefore, to confirm this hypothesis, measurements were performed evaluating the role of the DNP amount by changing its concentration (from 1.90 to 18.50 mg/L, pH 4.5, r.t) and fixing the size of CH/TiO_2_ at 1.0 × 2.0 cm. Figure 2B reports the obtained results. By increasing the DNP concentration, the adsorption capacities increased, but the plateau region was reached at a longer contact time, confirming the competition between DNP molecules for free active sites, retarding the removal [7]. However, the high DNP amount favored a greater concentration gradient between the bulk solution and the adsorbent surface, enhancing the pollutant mass transfer, especially at the beginning of the process, restituting higher q_t_ values [11,20]. In other words, the findings confirmed that the DNP mass transfer and its adsorption on the CH/TiO_2_ surface could have kinetic implications, as further demonstrated in the following sections.

### 2.4. Kinetic Analysis

Thanks to the kinetic analysis, additional information was obtained to obtain more insight into the process. The PFO (Equation (3)) and PSO (Equation (4)) kinetic models were applied to the experimental data. In particular, the attention was focused on q_t_ values from experiments during which the DNP concentration and the amount of the adsorbent were changed. Figure S2 reports the obtained kinetic evidence.

Specifically, in Figure S2A,B, the PFO model is reported to describe the data. Whereas in Figure S2C,D, the PSO model is applied. The R^2^ of each linear fitting and the outputted kinetic parameters are reported in Tables S1 and S2. The R^2^ values were high in both models, denoting that PFO and PSO could predict in synergy the process [7,32,33,35] with a great attitude of the PSO having R^2^ > 0.99 in each studied condition of work [7,32,33,35]. Accordingly, the comparison between the experimental adsorption capacities observed at the equilibrium, q_e,exp_, and those calculated, q_e,calc_, derived from the kinetic equation application, confirmed the finding: any important difference was observed between the application of PFO and PSO models. So, DNP removal onto CH/TiO_2_ was controlled both by the diffusion (PFO model) and adsorption (PSO model) mechanisms, as previously anticipated [20]. Regarding the role of diffusion, to infer more detailed information, the Weber–Morris equation was also applied to experimental data (Equation (5)). The model starts from the assumption that if the intraparticle diffusion is the only relevant kinetic step, the plot of q_t_ = f(t^1/2^) should return a straight line passing through the origin [7]. As reported in Figure 2C,D, multiple linear segments appeared necessary to describe the experimental data, denoting that the intraparticle diffusion was not the only key rate-determining step [7]. Specifically, two segments were used for fitting data points arising from experiments during which the amounts of DNP and adsorbent were changed. The first segment described the beginning of the process, and it was related to the kinetically important DNP diffusion from the bulk of the solution to the chitosan surface. Subsequently, at the end of the process, a second step corresponded to the intraparticle’s DNP diffusion and adsorption. Indeed, by extending the contact time, the DNP concentration in the solution decreased, and the q_t_ values tended to be leveled off. So, the adsorption process was hindered due to the minor availability of free active sites, and it acquired a kinetic relevance [7,32,33,35]. Moreover, by looking at the data reported in Figure 2C,D, it is possible to observe that the beginning of the second step was delayed when decreasing the adsorbent amount and increasing the DNP concentration. Hence, the findings well agree with the fact that when the number of free active sites was reduced with respect to the molecules of DNP, the intraparticle’s diffusion limiting step acquired a kinetic relevance [7,32,33,35].

### 2.5. Thermodynamic Analysis

**Figure 3:**
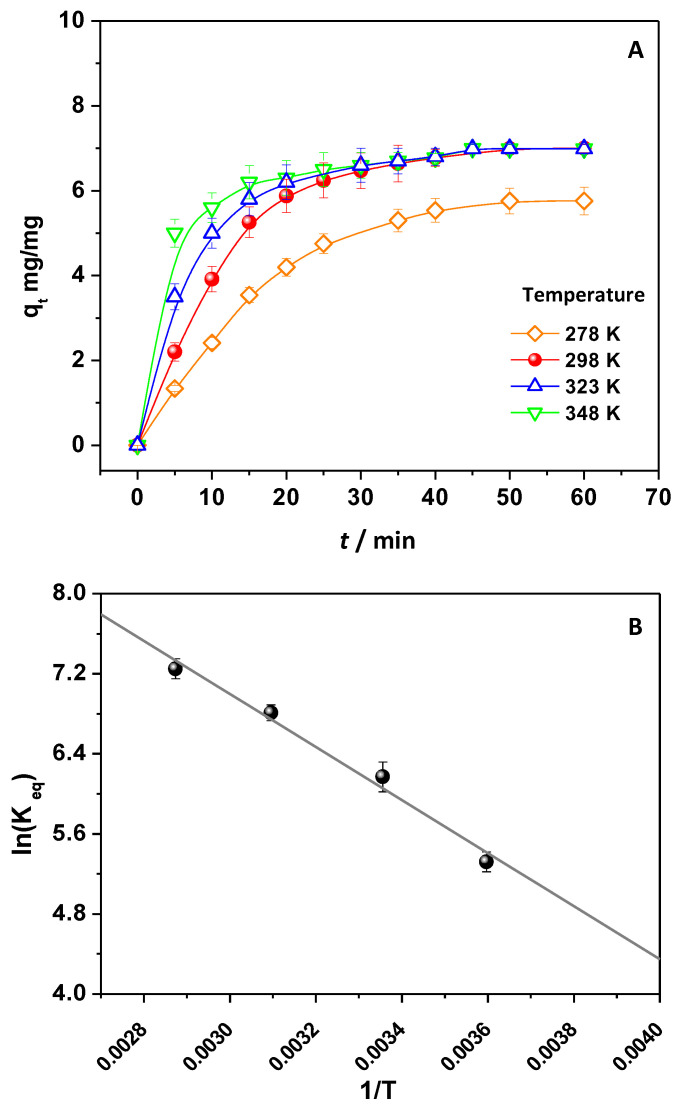
q_t_ values of CH/TiO_2_ calculated by changing the temperature values (**A**); a plot of ln(K_eq_) vs. 1/T to obtain ΔH° and ΔS° at 298 K (**B**).

The thermodynamic parameters of the process were inferred by changing the temperature of the solutions containing DNP. In detail, using a 1.0 × 2.0 cm CH/TiO_2_ and a DNP solution 18.50 mg/L solution at pH 4.5, the temperature was changed from 278 to 348 K. So, the q_t_ values were evaluated and reported in Figure 3A.

It was observed that the increase in temperature enhanced DNP adsorption, revealing the endothermic character. The q_t_ values increased, passing from 278 to 348 K, and the effect was pronounced, especially at the beginning of the process. Accordingly, since the DNP diffusion largely influenced the process, the rise in temperature should increment the diffusion rate of DNP, and, as expected, this effect was more pronounced at shorter contact time when free active sites were largely available onto the adsorbent surface. On this basis, the thermodynamic parameters were then calculated. The K_eq_ was determined, and for each value corresponding to different temperatures, the related ΔG° was inferred using Equation (6) (see Table S3). In detail, the calculated ΔG° values were negative and became more negative by increasing the temperature, indicating the spontaneous character of the investigated process [7,11,32,33,35]. By using Equations (7) and (8), ln(K_eq_) vs. 1/T (Figure 3B) was plotted. The ΔH°_298_ and ΔS°_298_ values were calculated (see Table S3) from the slope and intercept by applying a linear fitting. Both ΔH°_298_ (+22 ± 2 kJ/mol) and ΔS°_298_ (125 ± 1 J/mol × K) were positive, further confirming the endothermic character of the process and the increased randomness at the adsorbent–adsorbate interface, respectively [7]. Interestingly, another piece of evidence arose from the value of ΔH°_298_ < 40 kJ/mol, suggesting weak forces between the pollutant and adsorbent [7], whose nature is detailed in the following paragraph.

### 2.6. Effect of pH and Ionic Strength

**Figure 4:**
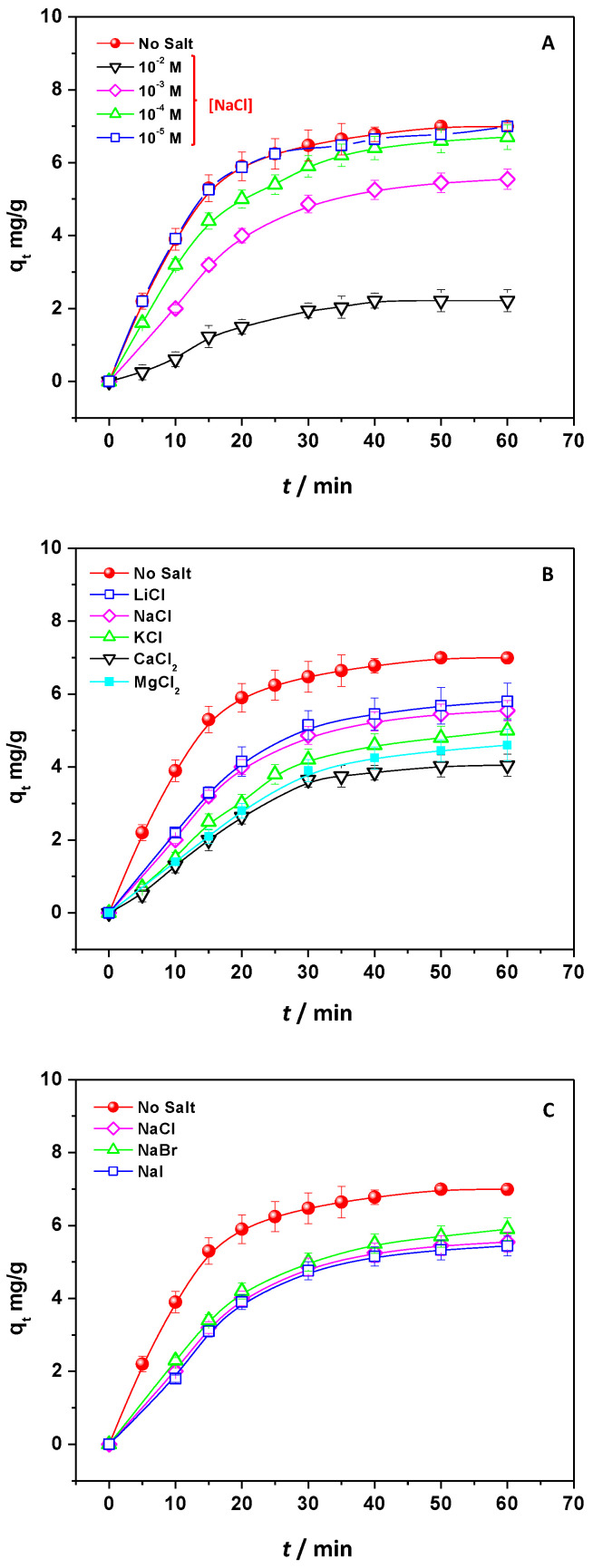
q_t_ values of CH/TiO_2_ calculated by changing the concentration of NaCl (**A**); by changing the nature of cation (**B**) and anion (**C**).

The pH effect on DNP removal was studied to detail the nature of the interaction between chitosan and DNP. For this purpose, 1.0 × 2.0 cm CH/TiO_2_ was swelled into a DNP solution 18.50 mg/L (at r.t), and the pH of that solution (pH = 4.5) was changed from 2 to 12 by using HCl or NaOH, according to the case. Since the pH of these solutions was significantly reduced at the extended contact time, the quantitative assessments, in terms of % of DNP adsorption, were evaluated in the first 10 min. This observation can be attributed to the CH/TiO_2′_s slight acidity, which, even if placed in pure water, decreased the solution pH to ~5 after 20 min of contact time. It is worth mentioning that the use of buffered solutions was excluded because, as will be discussed next, the ionic strength had a strong effect on the process. Consequently, DNP removal was evaluated by changing the pH values, and the results are reported in Figure S3. The % of adsorbed DNP was highest at pH 4.5, strongly lowering by the increase and decrease in the pH values. Comparing these data with similar studies [11,20], the presence of an important electrostatic interaction between DNP and CH/TiO_2_ was suggested. In detail, as reported in the inset of Figure S3, the adsorbent showed a PZC at around pH 7, suggesting that it was positively charged below this pH value (pH_PZC_) and negatively charged after pH_PZC_ [32,33,35], DNP molecules, instead, have a pK_a_ ≈ 4 [20] in water, corresponding to the loss of a proton from the alcoholic group. As a result, DNP can be found at pH < 4 as DNPH (not charged form) and at pH > 4 as DNP^−^ (anionic form). This information may account for the observed reduced adsorption, which can be attributed to the repulsion between the DNP and the adsorbent. At pH < 4, the adsorbent was positively charged, and DNP had no charge, so no attraction was observed. On the other hand, at 4 < pH < 7, the DNP was an anion, and the adsorbent was even positively charged, showing the strongest electrostatic attraction between them [7,20,32,33,35]. At pH > 7, both chitosan and DNP were negatively charged, and repulsion started to contribute. To further evidence the presence of weak and Coulombian interaction, experiments were performed in the presence of electrolytes at r.t and pH 4.5, when DNP was anion and chitosan was positively charged. According to the experimental conditions, if electrostatic interactions play a key role in the adsorption process, the electrolyte should screen the charges, thus reducing adsorption capacities. NaCl was chosen as the model salt for this assessment, and the different concentrations’ effects were evaluated during DNP adsorption. The corresponding q_t_ values were calculated and reported in Figure 4A. Particularly, it was observed that the NaCl inhibition effect started from a concentration of 1.0 × 10^−3^ M. The increase in NaCl amount reduced the q_t_ values, detecting a strong effect with NaCl 1.0 × 10^−2^ M. The results can be rationalized by considering (i) the competition effect of anions Cl^−^ for active sites onto chitosan and (ii) the interactions of cations with DNP^−^ that, by screening the charges, hindered the adsorption [7,32,33,35]. The nature of anions and cations was changed to individuate the main effect. By choosing 1.0 × 10^−3^ M as the salt reference concentration, the anion (Cl^−^) was fixed, and the cation was changed by adopting Li^+^, Na^+^, K^+^, Mg^2+^, and Ca^2+^. As reported in Figure 4B, increasing the cation size from Li^+^ to K^+^ decreased the q_t_ values, and the impact was significantly evident when K^+^ was in use. At the same time, the inhibiting effect was strongly evidenced by changing the charge of the cation, passing from monovalent to divalent one, by adopting for the comparison Ca^2+^ and Mg^2+^. The results can be rationalized by considering the radii of hydration of monovalent cations: K^+^ = 2.3, Na^+^ = 2.8, and Li^+^ = 3.4 Å [55]. Probably, K^+^, being the smallest hydrated ion, interacts strongly with DNP^−^, screening its charge and delaying the removal. The effect of bivalent cations arose from the presence of a double charge that, by increasing the ionic strength, largely reduced DNP adsorption [7,32,33,35].

Finally, the type of electrolytic anion was changed by fixing Na^+^ as a cation, and the effect of Cl^−^, Br^−^, and I^−^ was studied (Figure 4C). The q_t_ values, in this case, occurred similarly to each other, even if the anion nature changed. Since the effect of anion should probably be interpreted in terms of diffusion and competition onto the absorbent positively charged surface, no effect was observed in the explored contact time. On the other hand, the role of cations was directly exerted in the solution by screening the charge of the pollutant, and this effect occurred more pronounced during the process.

### 2.7. Swelling Ratio Measurements

The nature of the interaction between DNP and chitosan was a further object of investigation, and swelling measurements were carried out. Specifically, Equation (9) was used to infer the % of chitosan films swelling in the water medium, and a comparison between CH/TiO_2_ and CH/TiO_2_ + DNP was performed at different contact times (Figure S4). Indeed, the chitosan swelling in an aqueous medium is usually reported to be strongly affected by the protonation degree of chitosan amino groups [56]. As a whole, when chitosan film is placed in water at pH < pKa, chitosan (about pH = 6.5), the repulsion between these charges is usually observed, weakening the entanglement of the polymer chains, supporting the water channels formation in favor of the water uptake [56]. When referring to CH/TiO_2_, an expected high % of swelling in water was measured. Due to the slightly acidic pH (pH 5) of water acquired when in contact with the film, a % of chitosan swelling of 1600% was observed. While, if in the presence of the adsorbed DNP, although the pH of the DNP solution was 4.5, and the protonation of amino moieties should be at a larger extent, the % of chitosan film swelling at equilibrium collapsed at 1200%, indicating the acquired relative more hydrophobic character of the adsorbent [7,32,33,35]. This could be interpreted considering that chitosan’s positive charges are screened when the DNP- molecules are adsorbed (at pH 4.5) onto the film. So, the positively charged amino groups of the polymer are balanced, and the % of swelling of CH/TiO_2_ was reduced [56].

### 2.8. ATR-FTIR Measurements

To further detail the adsorption mechanism, ATR-FTIR measurements of CH/TiO_2_ were performed both before and after DNP adsorption. Specifically, in Figure S5, the typical chitosan spectrum can be observed when CH/TiO_2_ was investigated [22,23,24], denoting that the photocatalyst did not affect the main polymer features. Indeed, -NHR-CO- stretching was observed at 1644 cm^−1^, and, at 1550 cm^−1^, the signal ascribed both to the chitosan NH_2_ bending and the carboxylate stretching vibrations of acetate anions was observed. The bands at 1410 cm^−1^ and 1378 cm^−1^ confirmed the presence of acetate anions and a C-N bond, respectively. As expected, the C-O-C asymmetric and symmetric stretching appeared at 1151 cm^−1^ and 1066 cm^−1^, respectively. Finally, at 1025 cm^−1^, the C-O vibration of the alcoholic moieties was detected [22,23,24].

After this assessment, to compare CH/TiO_2_ with CH/TiO_2_+DNP, the ATR-FTIR spectrum of DNP was subsequently collected and reported in Figure S5, observing typical signals of phenols. Comparing the ATR-FTIR spectrum of the adsorbent before and after the addition of DNP, it is evident that the pollutant’s contribution was not significantly detectable due to the low amount of adsorbed DNP (Figure S6). On the other hand, slight changes were observed in the spectrum of chitosan and were diagnostic to unveil the mechanism of adsorption further.

Indeed, the CH/TiO_2_ signals in the wavenumber regions 1400–1800 cm^−1^ and 700–1200 cm^−1^ appeared affected when in the presence of DNP. The signals appeared broadening, with changes in the relative intensity ratio, and slightly shifted if compared with those referred to CH/TiO_2_. The findings confirmed, as in similar works [22,23,24], (i) the involvement of the chitosan amino groups during DNP adsorption due to electrostatic interaction, as discussed in the paper, and (ii) a DNP-induced hydrogen bonding reorganization, respectively.

### 2.9. Isotherms of Adsorption

**Figure 5:**
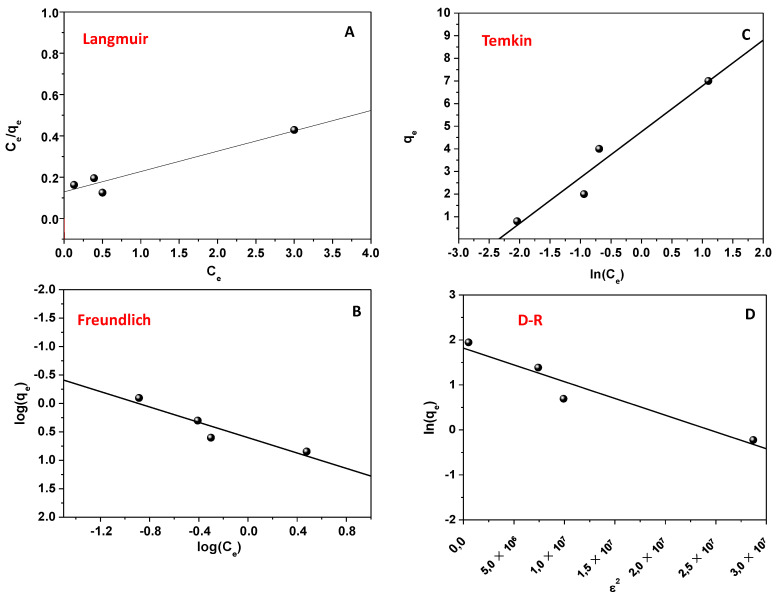
Isotherms of adsorption: Langmuir (**A**); Temkin (**B**); Freundlich (**C**); and D-R (**D**).

As usually reported in similar studies, the Langmuir, Freundlich, Temkin, and D-R isotherms were applied during this work. Figure 5 shows the obtained results. By focusing attention on experiments devoted to estimating the effect of DNP amount, different q_e_ and C_e_ values were thus obtained and correlated according to the isothermal models [7,35,54]. The data points were linearly fitted according to Equations (10)–(14). Therefore, the corresponding R^2^ values and the parameters that referred to each employed model were calculated and reported in Table S4. Due to the observed R^2^ value, the applicability of all the isotherms suggested that the process could be explained by considering more than a single mathematical model, underlining its heterogeneous character [7,35,54]. Therefore, DNP was adsorbed onto CH/TiO_2_ heterogeneous surfaces by forming multi-layers, and the heat of adsorption should change according to adsorbent–adsorbate interactions. When the D-R model was taken into account, other interesting information was obtained. The E values of 2.5 KJ/mol < 8 KJ/mol (calculated by using Equation (15)) confirmed that DNP adsorption involved the physisorption, and the result was consistent with the obtained ΔH°_298_ value [7,35,54]. The Q_0_ values arising from Langmuir and D-R models cannot be considered realistic due to the fact that the single model cannot describe the whole process. Indeed, these theoretical values are far from the experimental ones, which are 900 mg/g.

### 2.10. CH/TiO_2_ Regeneration

#### 2.10.1. Desorption of DNP and Reuse of the Adsorbent

**Figure 6:**
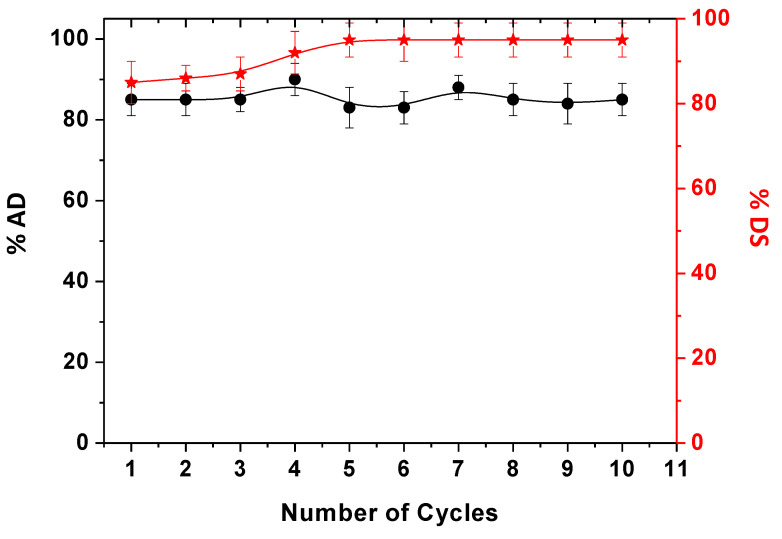
Consecutive cycles of DNP adsorption and desorption in 0.05 M NaCl. The black circles are referred to the % of adsorption; the red asterisks are referred to the % of desorption.

As discussed in the introduction, the main aim of this paper is the possibility of recycling the adsorbent. The first explored approach was focused on desorption experiments to recover even the pollutant. Due to the presence of Coulombian interactions between DNP and chitosan, a diluted NaCl solution (0.05 M) was selected to favor the adsorbent regeneration. Specifically, the adsorbent was swelled inside the salt-based solution after DNP adsorption and continuously stirred. For this purpose, different NaCl concentrations were tried, and the one that favored the quite complete desorption of DNP in a relatively short time was selected. So, the solution in contact with the chitosan film was spectrophotometrically monitored, and 20 min was selected as the best contact time for the complete desorption. Then, according to Equation (16), the % of desorbed DNP was calculated by evaluating the adsorbent reuse for 10 cycles of adsorption/desorption. The results, in terms of % of adsorption and desorption, are reported in Figure 6, denoting that the prolonged use of CH/TiO_2_ did not affect its efficiency, which remained high. Indeed, by looking at the error bars associated with each desorption measurement, it is possible to assess that significant statistical differences were not observed. Moreover, the slight increment of % DS after the fourth cycle could be associated with the retained pollutant in the previous cycles that was not completely desorbed.

#### 2.10.2. DNP Solid-State Photodegradation

The DNP solid-state photodegradation was studied as an alternative process to adsorbent recycling. Preliminarily experiments were thus performed to highlight another feature of the proposed adsorbent, worthy of investigation, opening novel horizons in this field. Equation (17) was used to infer the amount of DNP destroyed through photocatalytic processes. Indeed, the latter practices are well known in the literature [57] for water treatment; although, referring to DNP, the used AOPs were usually directly applied when DNP is dissolved in water [57,58,59,60]. AOPs were used during this work to assess the interesting possibility, not yet exploited for DNP, to favor the pollutant degradation when directly adsorbed onto the adsorbent. In this way, the suspension of the photocatalyst in water is avoided, not negatively perturbing the environment [39]. Moreover, at the same time, toxic light-induced by-products derived from DNP photodegradation should be retained onto CH/TiO_2_. In detail, the attention was focused on using UV, TiO_2_/UV, H_2_O_2_/UV, and TiO_2_/UV/H_2_O_2_. For comparison, experiments in the absence of TiO_2_ were also performed by using CH film and not CH/TiO_2_.

**Figure 7:**
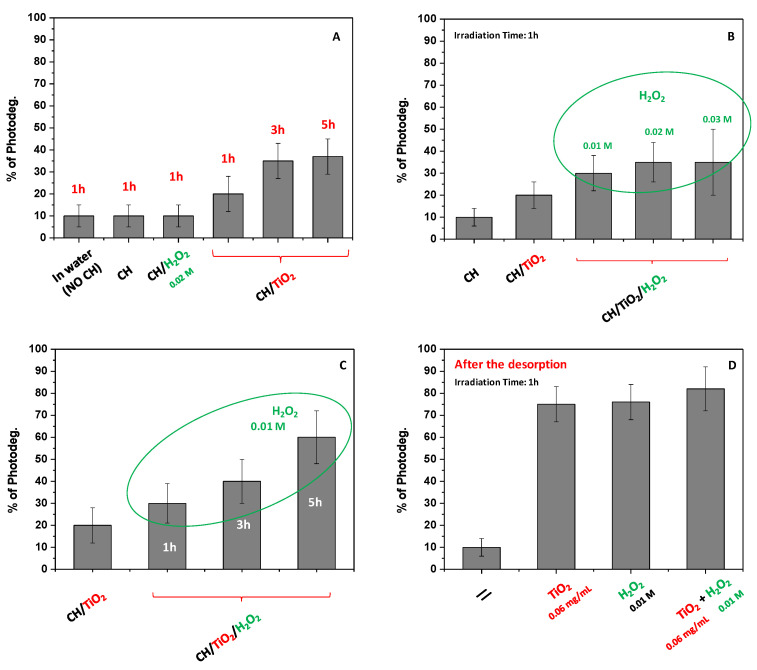
% of DNP photodegradation under different conditions of work: solid-state (**A**–**C**) and in-solution photodegradation (**D**). The irradiation time (in hours, h) is also indicated in each bar.

It is well known that, under the adopted work conditions, •OH radicals (highly reactive and not-selective) are the main produced species that favors DNP degradation [6]. To assess the latter purpose, as the first step, the role of simple UV radiation was evaluated by fixing 1 h as irradiation time. In this case, the film without TiO_2_ was adopted to adsorb DNP and then placed in pure water under continuous irradiation. As reported in Figure 7A, the % of photodegradation in 1 h was very low, obtaining only 10% of the DNP degradation. The result was also confirmed when the degradation was attempted directly in water containing DNP without CH, indicating that the DNP was relatively stable under UV light. The same result was also observed when H_2_O_2_ was added to the solution surrounding the CH film. Specifically, the concentration was fixed at 0.02 M, and no changes in terms of DNP degradation were observed with respect to only UV light. Conversely, the process enhancement was noted using the CH/TiO_2_ film. Indeed, the presence of TiO_2_ increased the DNP photodegradation, and the effect was more pronounced by extending the contact time from 1 to 5 h. In particular, the % of DNP degradation changed from 20% to 40%, ranging from 1 to 5 h, respectively. With the aim to improve the efficiency of the process but reduce the irradiation time at 1 h, the addition of H_2_O_2_ was considered, and its concentration was changed from 0.01 to 0.03 M, restituting a dose-dependent result (Figure 7B). Under these conditions, the DNP photodegradation increased up to 40%, even if irradiation time was settled at 1 h. The lack of important differences between the use of H_2_O_2_ at 0.02 and 0.03 M concentrations could be attributed to the hydroxyl radicals self-quenching in the solution surrounding the adsorbent [6]. Moreover, the TiO_2_ activation blocked onto CH could be reduced in favor of H_2_O_2_ photolysis, which acts as a UV “filter” when its concentration is high [6]. Overall, the combined use of H_2_O_2_ and TiO_2_ appeared useful, and a relatively low amount of H_2_O_2_ can be employed for the purpose. By extending the irradiation time to 5 h, the DNP photodegradation increases, reaching about 60% under this condition of work (Figure 7C). However, it is worth mentioning that using a greater H_2_O_2_ amount for an extended irradiation time favored the film’s degradation, which appeared solubilized in water. So, these harsh experimental conditions should be avoided if aiming to reuse the adsorbent again. While they should be useful to destroy both the adsorbent and adsorbate.

Finally, a valid alternative to the proposed approaches could be the combination of the described two methods for obtaining the adsorbent regeneration and the pollutant degradation. In particular, the first step is pollutant desorption, which can be subsequently subject to its well-known photodegradation in water. In this case, as reported in Figure 7D, the use of 0.06 mg/mL of TiO_2_ was enough to induce almost complete DNP degradation that, if compared with the use of only UV light, appeared ~8 times greater. When H_2_O_2_ was used alone, similar results were retrieved, and the DNP photodegradation that occurred, to some extent, improved when TiO_2_ and H_2_O_2_ were combined in use.

## 3. Materials and Methods

### 3.1. Chemicals

The chemicals used were of analytical grade, and samples were prepared using deionized water. Commercial grade chitosan powder (from crab shells, highly viscous, with deacetylation degree ≥ 75%, average M.W. = 150 kDa), acetic acid (99.9%), glycerol (+99.5%), and DNP (C_6_H_4_N_2_O_5_, MW 184.106 g × mol^−1^) were purchased from Sigma Aldrich (Milan, Italy). DNP stock solution with a concentration of 100 mg × L^−1^ was prepared. According to the case, the pH of the DNP solutions was modified by adding HCl and NaOH. On the other hand, salts such as NaCl, LiCl, KCl, MgCl_2_, CaCl_2_, NaBr, and NaI (Sigma Aldrich, Milan, Italy) were employed to investigate the ionic strength role during the adsorption. Aeroxide TiO_2_ P25 was purchased from Evonik Industries AG and used as received. The measurements were performed in triplicate, and error bands were reported as standard deviations.

### 3.2. Preparation of Chitosan/TiO_2_ Films

Chitosan (CH) was solubilized in water to obtain a concentration of 1% (*w*/*v*) in presence of acetic acid 0.8% (*v*/*v*). The mixture was continuously stirred for 24 h to ensure complete chitosan solubilization. Glycerol was added (200 μL/100 mL) to the mixture as a plasticizer. The obtained hydrogel was filtered and degassed for 1 h. Then, TiO_2_ (50 mg) was dispersed inside the chitosan hydrogel, sonicating and stirring for 1 h to ensure a homogeneous distribution. Plastic Petri plates were used as a template for forming the solid-state substrates. Specifically, the obtained hydrogel was placed inside the plates and maintained in an oven at 60 °C for 24 h. The water evaporation occurred by rearranging chitosan chains to form free-standing CH/TiO_2_ blended films.

### 3.3. UV–Visible Measurements

The Varian CARY 5 UV–Vis–NIR spectrophotometer (Varian Inc., now Agilent Technologies Inc., Santa Clara, CA, USA) was employed to register the visible absorption spectra of DNP in a range of 200–800 nm at a scan rate of 1 nm/s. The DNP concentration was calculated by measuring the absorbance intensity at λ 358 nm and adopting a molar absorption coefficient (ε) of 0.041 L × mg^−1^ × cm^−1^ derived from the application of the Lambert–Beer law. Diffuse Reflectance Spectroscopy (DRS) measurements were also performed by using an appropriate tool, to evaluate the % of Reflectance (% R) of samples.

### 3.4. Scanning Electron Microscopy (SEM) Investigation

The surface morphology of the CH/TiO_2_ composite films was investigated using an electron microscope FESEM-EDX Carl Zeiss Sigma 300 VP. The samples were fixed on aluminum stubs and then sputtered with graphite using a Sputter Quorum Q150. Additionally, the chemical composition was determined by EDX under the scanning electron microscope and X-ray diffraction.

### 3.5. ATR-FTIR Spectroscopy Measurements

ATR-FTIR analyses were performed on chitosan film before and after DNP adsorption. The spectra were recorded in a 600–4000 cm^−1^ range, at a resolution of 4 cm^−1^, using a Fourier Transform Infrared spectrometer 670-IR equipped with an ATR device (Varian Inc., now Agilent Technologies Inc., Santa Clara, CA, USA). A total of 32 scans were summed for each acquisition.

### 3.6. In Batch Adsorption Experiments

After placing in contact DNP solution with the adsorbent, under constant stirring, the % of pollutant adsorption, % AD, was inferred by using Equation (1) [7,35]:

(1) $$\%AD=\frac{C_{0}-C_{t}}{C_{0}}\times 100$$

with C_0_ and C_t_ representing the concentration of the investigated DNP solution, measured at λ 358 nm, at time t_0_ and *t* time, respectively.

The adsorption capacities q_t_ (mg × g^−1^) were inferred from Equation (2) [7,35]:

(2) $$q_{t}=\frac{C_{0}-C_{t}}{W}\times V$$

V is the volume of water (15 mL), W is the amount of dried film (g), and C_0_ and C_t_ are the DNP concentrations (mg/L) calculated at time t_0_ and *t* time, respectively.

The study was first performed by evaluating the effect of the CH/TiO_2_ and DNP amount on adsorption.

Specifically, to investigate the DNP role, a film having a size of 1.0 × 2.0 cm (0.033 g) was placed into the water properly polluted with DNP added in different concentrations ranging from 1.90 to 18.50 mg/L at pH 4.5 (that is the pH of DNP in water). On the other hand, to assess the adsorbent amount role, its size, and thus its weight was changed from 0.5 × 1.0 to 2.0 × 2.0 cm (from 0.009 to 0.070 mg) in the presence of DNP 18.50 mg/L. The process was studied under constant stirring at room temperature (298 K), continuously monitoring the pH of the solutions. Experiments to study the role of different parameters (such as ionic strength, pH, and temperature) affecting DNP removal were performed by fixing the amount of the adsorbate at 18.50 mg/L and adsorbent at 0.033 g (1.0 × 2.0 cm), respectively.

### 3.7. Adsorption Kinetics

The pseudo-first-order (PFO) and pseudo-second-order (PSO) kinetics models, described by Equations (3) and (4), respectively, were adopted [7,35,61]. Specifically, experiments referred to the change in adsorbent and adsorbate amounts were subjected to this investigation.

(3) $$\ln\left(q_{e}-q_{t}\right)=\ln\left(q_{e}\right)-K_{1}\times t$$

(4) $$\frac{t}{q_{t}}=\frac{1}{K_{2}q_{e}^{2}}+\frac{1}{q_{e}}\times t$$

q_e_ (mg/L) represents the adsorption capacities at equilibrium, and q_t_ (mg/L) is the adsorption capacity at time *t*. K_1_ (min^−1^) and K_2_ (g/(mg×min)) are the PFO and PSO rate constants, respectively.

The intraparticle diffusion role was also explored, and the Weber–Morris model was applied to experimental data (Equation (5)) [7].

(5) $$q_{t}=k_{int}\times t^{1/2}+C$$

k_int_ represents the kinetic constant expressed in mg/(g × min^1/2^), referred to intraparticle diffusion rate, and C is the thickness of the boundary layer.

### 3.8. Thermodynamic Study

Adsorption experiments were accomplished at different temperature values ranging from 278 to 348 K. The adsorption of DNP was thus studied, and the q_t_ values were inferred until the equilibrium was reached. The ΔG° was calculated by using Equation (6) [6,7]:

ΔG° = − RT ln K_eq_ (6)

R is the universal gas constant (8.314 J/(mol × K)), T is the temperature (K), and K_eq_ represents the equilibrium constant expressed as q_e_/C_e_. By combining these, Equations (6)–(8) were obtained and used to calculate ΔH° and ΔS° [7].

ΔG° = ΔH° − TΔS° (7)

(8) $$lnK_{eq}=-\frac{{∆H}^{0}}{RT}+\frac{{∆S}^{0}}{R}$$

### 3.9. Swelling Ratio Measurements

The swelling of the adsorbent in water when in the absence and presence of DNP was measured. For this purpose, a 1.0 × 2.0 cm film was swelled in bi-distilled water at controlled room temperature, r.t., (298 K). Measurements were performed until the equilibrium was attained, so the films were blotted with filter paper and weighed. Equation (9) was used to express the % of swelling (% S): [56]

(9) $$\%S=\frac{W_{s}-W_{d}}{W_{d}}\times 100$$

where W_s_ is the weight of the film swelled in water at time *t*, and W_d_ is the weight of the dried film.

### 3.10. Determination of Chitosan/TiO_2_ Film Zero-Point Charge

The zero-point charge pH (pH_ZPC_) of the CH/TiO_2_ was evaluated. In detail, 5.0 × 10^−2^ M NaCl solutions (30 mL) were prepared, and the corresponding pH values were adjusted from 2 to 12 (pH_i_). Subsequently, CH/TiO_2_ (0.033 g corresponding to a film size of 1.0 × 2.0 cm) was placed inside these solutions and stirred at 298 K for 48 h. The final pH (pH_F_) values were then measured. The plot of pH_i_ vs. pH_F_ values was obtained to infer the pH_ZPC_ [33,35,62].

### 3.11. Isotherms of Adsorption

The results obtained during the investigation of the DNP amount effect were used for pursuing the study. Particularly, the obtained q_e_ and C_e_ values were employed according to the Langmuir, Freundlich, Temkin, and Dubinin–Radushkevich isotherms (Equations (10)–(13)) of adsorption [7,54]. These models were largely applied in the literature for this kind of study, offering interesting information about adsorption [7,54]. Specifically, the Langmuir model (Equation (10)) describes a process in which the used adsorbent has all the adsorption sites characterized by constant energy. Furthermore, an adsorbed monolayer in which the pollutant interacts only with the substrate is also supposed. The interaction between the contaminant molecules is excluded.

(10) $$\frac{C_{e}}{q_{e}}=\frac{1}{K_{L}Q_{0}}+\frac{C_{e}}{Q_{0}}$$

q_e_ (mg/g) is the adsorption capacity of CH/TiO_2_ at equilibrium, and C_e_ is the correspondent equilibrium concentration in solution expressed in mg/L. The Langmuir equilibrium constant is K_L_ (L/mg), and the adsorbent maximum adsorption capacity (mg/g) value is Q_0_.

With respect to the previous model, the Freundlich isotherm (Equation (11)) [7,54] refers to the adsorption onto a heterogeneous material with adsorption sites having different energies. In particular, the energy involved during the adsorption decreases exponentially during the pollutant removal.

(11) $$log\left(q_{e}\right)=log\left(K_{F}\right)+\frac{1}{n}log\left(C_{e}\right)$$

If the Freundlich constant is K_F_ (L/mg), the value of n reflects the character of the process: a value of 1/n = 0 suggests that the adsorption process is irreversible. On the other hand, values of 1/n > 1 and 0 < 1/n < 1 indicate that the process is favorable and unfavorable, respectively.

As for the latter model, the application of the Temkin isotherm (Equation (12)) [7,54] suggests that the heat of adsorption changes during the process, but it decreases linearly during the removal due to adsorbent/adsorbate interactions.

(12) $$q_{e}=B_{1}ln\left(K_{T}\right)+B_{1}ln\left(C_{e}\right)$$

K_T_ (L/mol) and B_1_ refer to the equilibrium binding constant and the heat of adsorption, respectively.

Finally, the Dubinin–Radushkevich (D-R) isotherm model was adopted (Equation (13)) [7,54]. The model reports that the adsorption occurs on a heterogeneous surface having a Gaussian energy distribution.

(13) $${lnq}_{e}=ln(Q_{0})-K_{D-R}\times \varepsilon^{2}$$

q_e_ (mg/g) is the equilibrium adsorption capacity, Q_0_ (mg/g) is the theoretical maximum adsorption capacity, and K_D-R_ (mol^2^/J^2^) is the Dubinin–Radushkevich isotherm constant. ε is the Polanyi potential, and it is described by Equation (14).

(14) $$\varepsilon =RTln(1+\frac{1}{C_{e}})$$

R is the gas constant (8.314 J/(mol K)), T is the absolute temperature (K), and C_e_ represents the pollutant equilibrium concentration (mg/L) [7,33]. By knowing the K_D-R_ value, Equation (15) [7,54] was used to calculate the energy value E involved during the process. Information about the nature of the adsorption, distinguishing between physical and chemical interaction, can be thus inferred.

(15) $$E=\frac{1}{\sqrt{2K_{D-R}}}$$

Indeed, if E < 8 kJ/mol, the pollutant physisorption should be mainly considered. On the other hand, chemisorption occurs for values E > 8 kJ/mol [7,54].

### 3.12. CH/TiO_2_ Regeneration: In Batch Desorption Experiments

After DNP adsorption from water, a water NaCl solution of 0.05 M was adopted to release the pollutant for regenerating the adsorbent. UV–Vis spectroscopy was used to infer the desorbed DNP amount. Specifically, the adsorbent was first removed from polluted water after 60 min, adopted as contact time, and washed with fresh water to avoid the interference of the not adsorbed DNP. Then, it was swelled for 20 min in a NaCl solution for pollutant release. The regenerated adsorbent was used for 10 adsorption and desorption cycles by following the procedure detailed so far. The % of desorption, % DS, was calculated according to Equation (16).

(16) $$\%DS=\frac{C_{rel}}{C_{ads}}\times 100$$

with C_rel_ = DNP concentration obtained after the release in NaCl, while C_ads_ = amount of adsorbed DNP.

### 3.13. CH/TiO_2_ Regeneration: Solid-State Photocatalytic Experiments

The solid-state photodegradation of DNP was performed by employing AOPs. A UV lamp (UV fluorescent lamp, Spectroline, Model CNF 280 C/FE, λ 254 nm, light flux 0.2 mW/cm^2^; Melville, NY, 11747, USA) was used to irradiate the adsorbent. Once again, the adsorbent was first removed from polluted water after 60 min, adopted as contact time, and washed with fresh water to avoid the interference of the not adsorbed DNP. Then it was placed in freshwater (15 mL) and exposed to UV light. Experiments were carried out to evaluate the role of irradiation time and the amount of H_2_O_2_ (stock solution 30%) purposely added to the water solution containing the adsorbent. The % of the solid-state photodegraded DNP was calculated by performing desorption experiments after the irradiation. By knowing the amount of adsorbed DNP, and thus the % of desorption in the absence of irradiation (Equation (16)), the % of photodegradation (% P) was calculated by using Equation (17).

(17) $$\%P=\frac{C_{rel}-C_{rel\prime }}{C_{ads}}\times 100$$

where C_rel_ and C_rel’_ represent the DNP concentration obtained after the release in NaCl without and with light irradiation, respectively, while C_ads_ represents the amount of adsorbed DNP.

## 4. Conclusions

This work reported the use of chitosan-based films for DNP adsorption from water, showing a maximum adsorption capacity of 900 mg/g. The TiO_2_ photocatalyst was blended inside the chitosan network to give it photocatalytic properties, thus favoring the solid-state photodegradation of the pollutant for finally regenerating the adsorbent substrate. Indeed, the regeneration and reuse of the adsorbent were also studied, and for this purpose, a valid alternative considered was the DNP desorption through a diluted NaCl solution. DNP was completely adsorbed and quickly desorbed, enabling the possibility of 10 cycles of adsorption/desorption being performed, which potentially could be increased. On the other hand, the use of AOPs favored chitosan reuse, not permitting DNP recovery. In detail, the processes were fully investigated using UV–Vis spectroscopy, monitoring the main absorption band of DNP and its time evolution. The adsorption was studied by changing several operational parameters, and it was observed that the pollutant removal from water increased by increasing the temperature values. The process was spontaneous (ΔG° < 0) and endothermic (ΔH° > 0), occurring with an increase in entropy. Moreover, the chitosan adsorption capacities increased by increasing the adsorbent and pollutant amount, suggesting the important role of free active sites on the chitosan surfaces and DNP diffusion during the adsorption. The presence of electrostatic interaction between the pollutant and the adsorbent was confirmed by changing the pH values and ionic strength of solutions containing DNP. The results were unveiled by swelling and ATR-FTIR measurements. The isotherm and the kinetic models were finally studied, evidencing the heterogeneous character of the process that could be described by adopting PFO and PSO kinetic models.

## Figures and Tables

**Table 1:** Maximum adsorption capacities (q_max_) values comparison between several adsorbents already described in the literature for DNF removal, compared with Chitosan/TiO_2_ composite film proposed in the present work.

Adsorbent	q_max_ (mg/g)	Reference
**Active carbon**	277.78	[14]
**SiO_2_**-based nanocomposite	Not available. % removal: 58.66% at pH 6.4	[15]
Amine-functionalized metal-organic framework	Not available. % removal: 99% at pH 4	[16]
Ultrasound-assisted magnetic adsorption graphene oxide-Fe_3_O_4_-based system	425.58	[17]
A polymer obtained by loading ionic liquids on silica	114.7	[8]
Sol–gel Titania-silica-mixed imidazolium-based ionic liquid	7.78	[18]
Char ash	7.55	[19]
Chicken manure biochar	148.1	[20]
***Chitosan/TiO_2_ film***	*900*	*This work*

**Table 2:** Some interesting examples referred to TiO_2_-based materials described in more recent literature for the adsorption and photodegradation of DNP.

Adsorbent	Reference
Water-compatible molecularly imprinted thiol-functionalized activated titanium dioxide	Zhou et al. [25]
TiO_2_/activated carbon	Cao et al. [26]
Multi-walled carbon nanotubes (MWCNTs)/TiO_2_ composite	Wang et al. [27]
Fe_3_O_4_@SiO_2_@TiO_2_/rGO magnetic nanoparticles	Hedayat et al. [28]
Fe_3_O_4_ nanoparticles using Chlorella vulgaris extract	Al Garni et al. [29]
Flexible hollow TiO_2_@CMS/carbon-fiber van der Waals heterostructures	Chen et al. [30]
Ag_2_CO_3_-loaded phosphorus and sulfur co-doped graphitic carbon nitride nanosheets	Raizada et al. [31]

## Data Availability

Not applicable.

## Supplementary Materials

The following supporting information can be downloaded at: https://www.mdpi.com/article/10.3390/ijms24108552/s1.
