# Supplementary material for: The Adsorption Efficiency of Regenerable Chitosan-TiO2 Composite Films in Removing 2,4-Dinitrophenol from Water
Source: Int J Mol Sci. 2023 May 10;24(10):8552. doi: 10.3390/ijms24108552 (PMC10218325; doi:10.3390/ijms24108552)
Supplement: Supplementary file 1 [file ijms-24-08552-s001.zip › ijms-2354604-supplementary.pdf]

ELECTRONIC SUPPORTING INFORMATION (ESI)

The Adsorption efficiency of regenerable Chitosan-TiO_2 composite films in removing 2,4-Dinitrophenol from water

Jennifer Gubitosa[a], Vito Rizzi[a*], Paola Fini[b], Sergio Nuzzo[b], Pinalysa Cosma[a,b*]

[a]Università degli Studi di Bari "Aldo Moro", Dipartimento di Chimica, Via Orabona, 4-70126 Bari, Italy

[b]Consiglio Nazionale delle Ricerche CNR-IPCF, UOS Bari, Via Orabona, 4-70126 Bari, Italy

*Corresponding Authors: Prof. Pinalysa Cosma:

Università degli Studi di Bari "Aldo Moro"

Dipartimento di Chimica, Via Orabona 4-70126 Bari, Italy

Tel: +390805443443

E-mail pinalysa.cosma@uniba.it

*Dr. Vito Rizzi:

Università degli Studi di Bari "Aldo Moro"

Dipartimento di Chimica, Via Orabona 4-70126 Bari, Italy

Tel: +390805443443

E-mail vito.rizzi@uniba.it

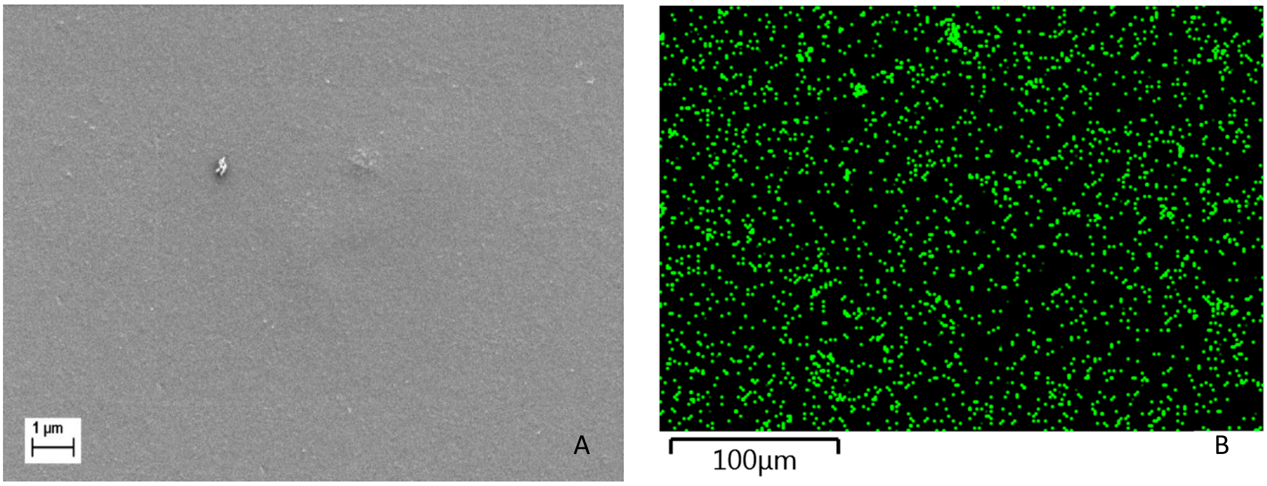

Figure S1: SEM image of CH/TiO_2 (**A**); EDS analysis of CH/TiO_2 (**B**)

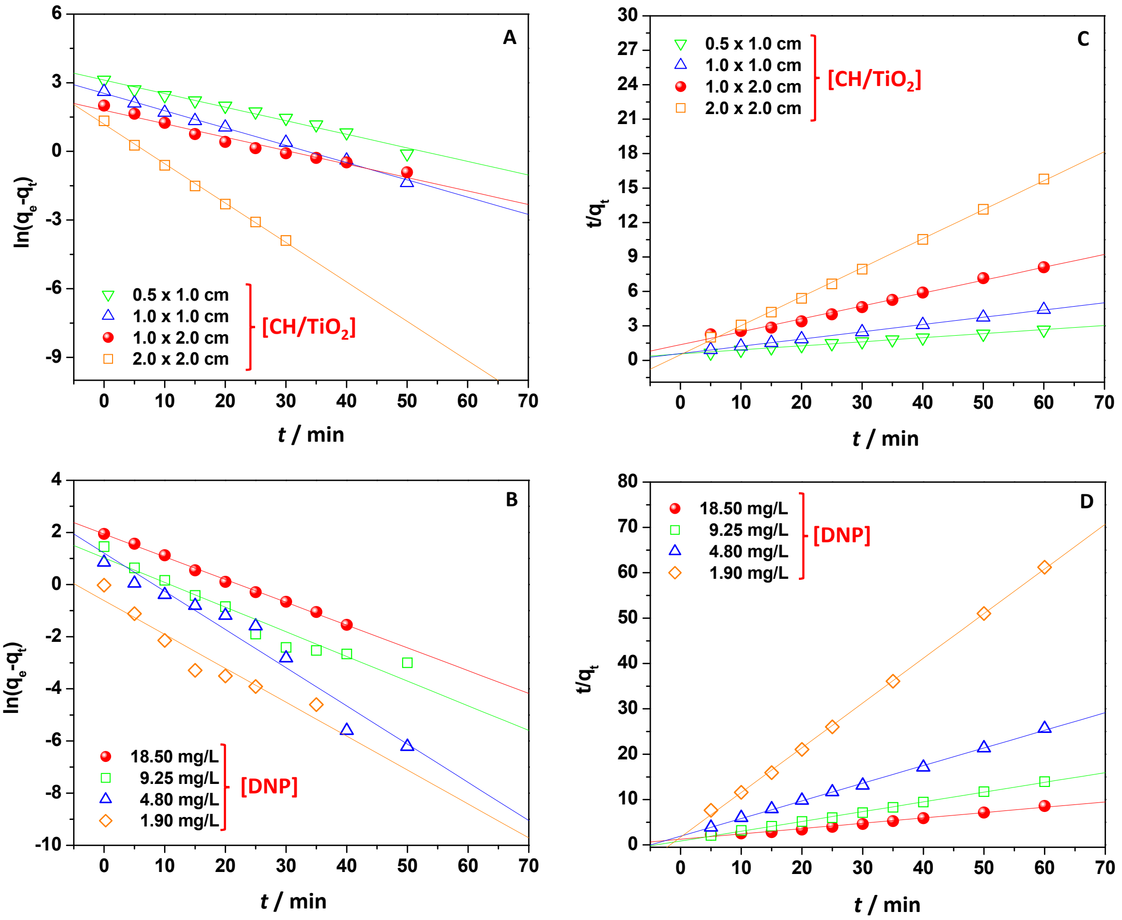

Figure S2: Pseudo-first (**A**, **B**) and Pseudo-second (**C**, **D**) order kinetic models applied to experimental data in which the amounts of adsorbent and adsorbate are changed.

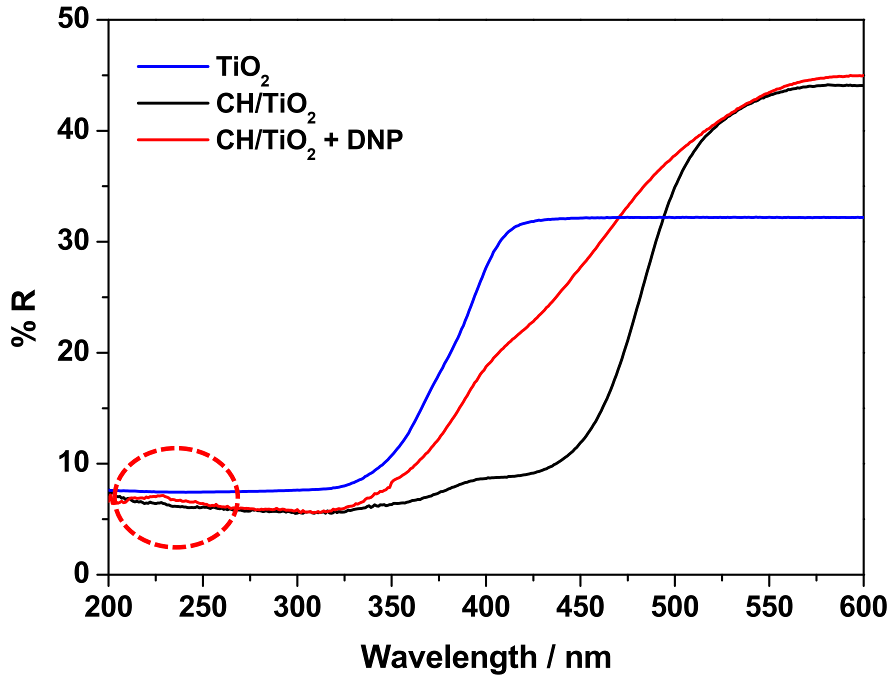

Figure S3: DRS of TiO_2 in powder, CH/TiO_2 and CH/TiO_2 + DNP.

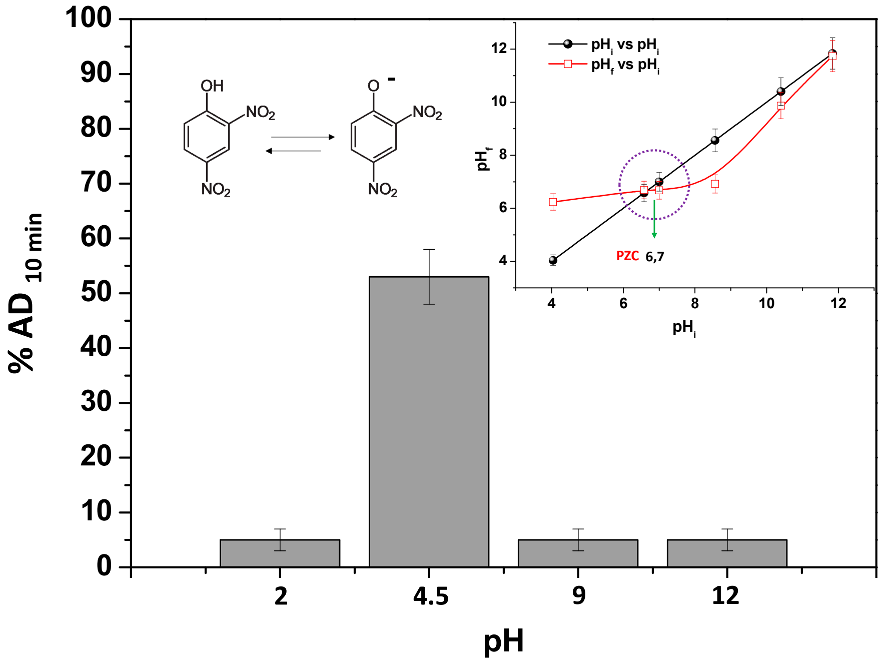

Figure S4: % of DNP adsorption onto CH/TiO_2 at different pH values. The *inset* reports the method to infer the pH_{PZC}.

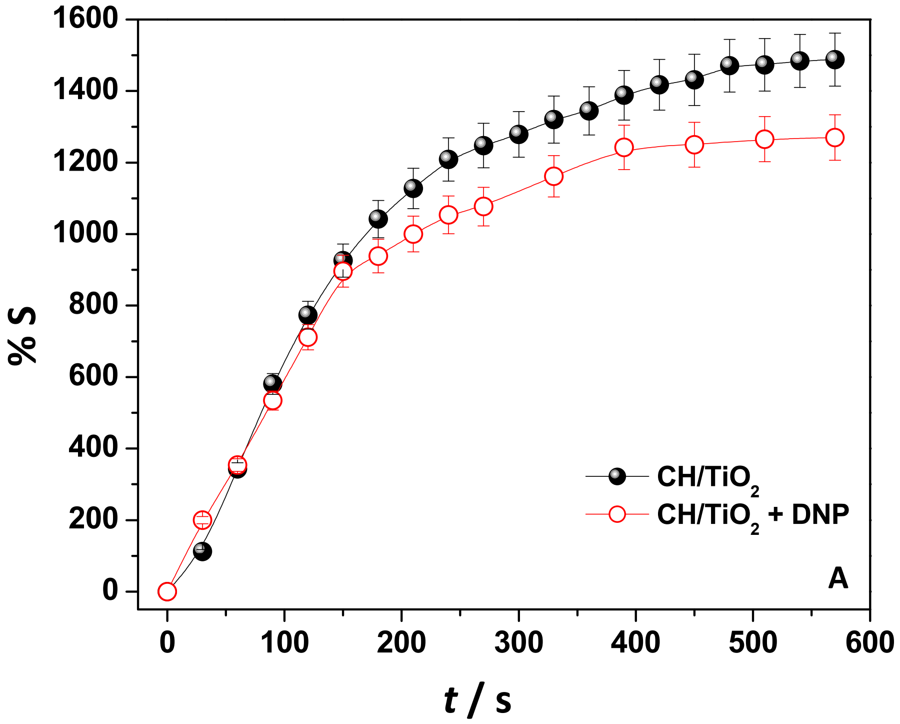

Figure S5: % of CH/TiO_2 and CH/TiO_2 + DNP swelling in water.

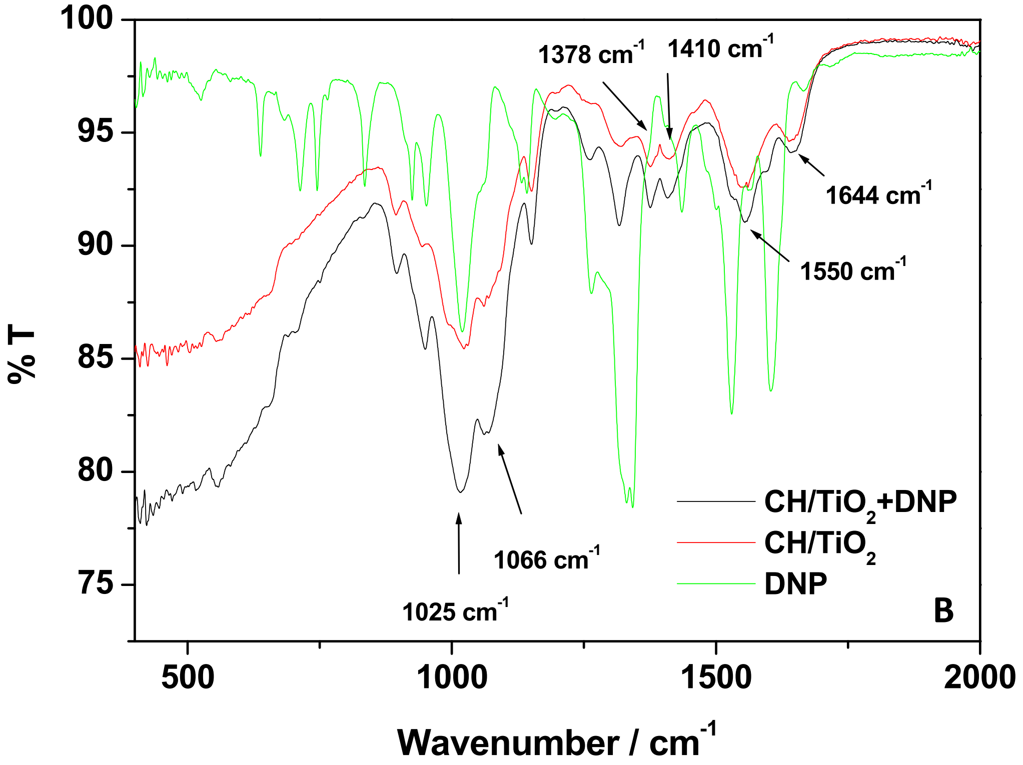

Figure S6: ATR-FTIR spectra of CH/TiO_2 in presence and absence of DNP.

Concentration (DNP) mg/L	Pseudo first-order				Pseudo second-order			
	q_e exp mg/g	q_e calc mg/g	K_1 1/min	R^2	q_e exp mg/g	q_e calc mg/g	K_2 g/mg×min	R^2
18.50	7	7	6×10^{-2}	0.9855	7	8	1×10^{-2}	0.9961
9.25	4	3	9×10^{-2}	0.9668	4	5	5×10^{-2}	0.9992
4.80	2	3	2×10^{-1}	0.9746	2	3	8×10^{-2}	0.9993
1.90	0.8	2	1×10^{-1}	0.9571	0.8	1	5×10^{-1}	0.9996

Table S1: Kinetic parameters referred to experiments in which the DNP amount is changed.

Chitosan size (cm×cm) and amount (g)	Pseudo first-order				Pseudo second-order			
	$q_e$exp mg/g	$q_e$calc mg/g	K_1 1/min	R^2	q_e exp mg/g	q_e calc mg/g	K_2 g/mg×min	R^2
0.5 × 1.0 (0.009 g)	24	23	6×10^{-2}	0.9915	24	27	2×10^{-3}	0.9981
1.0 × 1.0 (0.018 g)	14	13	8×10^{-2}	0.9960	14	17	7×10^{-3}	0.9999
1.0 × 2.0 (0.033 g)	7	7	6×10^{-2}	0.9855	7	8	1×10^{-2}	0.9961
2.0 × 2.0 (0.070 g)	4	3	2×10^{-1}	0.9987	4	4	1×10^{-1}	0.9995

Table S2: Kinetic parameters referred to experiments in which the adsorbent amount is changed.

$\Delta H°_{298K}$ KJ/mol	$\Delta S°_{298K}$ J/(mol×K)	$\Delta G°_{278K}$ KJ/mol	$\Delta G°_{298K}$ KJ/mol	$\Delta G°_{323K}$ KJ/mol	$\Delta G°_{348K}$ KJ/mol
22±2	125±1	-12±2	-15±1	-18±4	-21±2

Table S3: Thermodynamic parameters

**Freundlich Isotherm Model**			**Temkin Isotherm Model**			
**K_F (L/mg)**	**n**	**R^2**	**K_T (L/mol)**	**B_1**	**R^2**	
**4**	**1.50**	**0.9392**	**10**	**2**	**0.9730**	
**Lngumir Isotherm Model**			**D-R Isotherm Model**			
**K_L (L/mg)**	**Q_0 (mg/g)**	**R^2**	**K_{D-R} (mol^2/J^2)**	**Q_0 (mg/g)**	**E(KJ/mol)**	**R^2**
**1**	**10**	**0.9633**	**8×10^{-8}**	**6**	**2.5**	**0.9610**

Table S4: Isotherms parameters
